# Continued cytoadherence of *Plasmodium falciparum* infected red blood cells after antimalarial treatment

**DOI:** 10.1016/j.molbiopara.2009.09.007

**Published:** 2010-02

**Authors:** Katie R. Hughes, Giancarlo A. Biagini, Alister G. Craig

**Affiliations:** Liverpool School of Tropical Medicine, Pembroke Place, Liverpool, L3 5QA, UK

**Keywords:** iRBCs, infected red blood cells, PfEMP-1, *Plasmodium falciparum* erythrocyte membrane protein 1, DBL, Duffy binding like, ICAM-1, intercellular adhesion molecule 1, CSA, chondroitin sulphate A, HUVEC, human umbilical vein endothelial cells, HDMEC, human dermal microvascular endothelial cells, TNF, tumour necrosis factor, FACS, fluorescence activated cell sorter, Malaria, Endothelial, Pathogenesis, Adhesion, ICAM-1, Artemisinin, Quinine

## Abstract

Development of severe disease in *Plasmodium falciparum* malaria infection is thought to be, at least in part, due to the sequestration of trophozoite-stage infected red blood cells in the microvasculature. The process of cytoadherence is mediated by binding of the parasite protein PfEMP-1 on the surface of infected red blood cells to endothelial cell receptors. Although antimalarial treatments rapidly kill parasites, significant mortality is still seen in severe malaria, particularly within 24 h of hospital admission. We find that cytoadherence of infected red blood cells continues for several hours after killing of the parasite by antimalarials; after 24 h treatment using a range of antimalarials binding is approximately one-third the level of untreated parasite cultures. This is consistent with the maintained presence of PfEMP-1 on the surface of drug-treated infected red blood cells. A specific advantage of artesunate over other treatments tested is seen on addition of this drug to younger ring stage parasites, which do not mature to the cytoadherent trophozoite-stage. These findings show that cytoadherence, a potential pathogenic property of *P. falciparum* infected red blood cells, continues long after the parasite has been killed. These data support the development of adjunctive therapies to reverse the pathophysiological consequences of cytoadherence.

## 1 Introduction

*Plasmodium falciparum* malaria is responsible for over a million deaths a year [1] with many of these fatalities occurring in infants in Africa. An important aspect of the pathogenesis of severe malaria results from the ability of infected red blood cells to sequester in the microvasculature. During the 48 h parasite growth stage inside a host red blood cell the parasite makes many changes to the cell. As well as becoming more metabolically active, and more rigid, the infected red blood cell becomes capable of adhering to the endothelial cells lining the blood vessels [2]. Post-mortem studies of severe malaria show high levels of infected red blood cells (iRBCs) bound to microvasculature [3,4]. The involvement of sequestration in pathogenesis could be directly a result of blocking of the blood vessels, and/or downstream effects caused by the interaction between iRBC and the endothelium, including local inflammatory responses [5].

Cytoadherence is mediated by a parasite protein PfEMP-1 which is a large protein comprising several external Duffy-binding-like (DBL) domains, encoded for by *var* genes. *P. falciparum* has around 60 *var* genes each encoding a PfEMP-1 protein that is probably antigenically distinct and may have different cytoadherent properties. In vitro studies have suggested that each parasite expresses just one of the 60 PfEMP-1 proteins at a time, although the situation is less clear in vivo. Switching expressed *var* gene and hence PfEMP-1 protein, the process of antigenic variation, is a way in which the parasite may be able to evade recognition by host adaptive antibody immune responses. In addition, there is a large variation in *var* gene repertoire between individual parasites. The large number of variants of PfEMP-1 also results in the potential to bind to many different host receptors [6].

Of the 14 cell adhesion receptors to which iRBC can bind, a few have been well characterized including ICAM-1, CD36 and chondroitin sulphate A (CSA). ICAM-1 is of interest as it has been linked to cerebral malaria [7]. Patients suffering with cerebral malaria have up-regulated levels of ICAM-1 in the brain and parasite isolates from patients with malaria disease (including those with CM and compared to asymptomatic infection) showed higher binding to ICAM-1 protein [8,9]. Although other studies have not observed such a link [10,11], the ability to adhere to ICAM-1 has been observed in a significant proportion of patient isolates [11,12], and the adhesion interaction has been well studied [13–15]. CD36 is expressed on many types of endothelia (but negligibly in the brain) and has the ability to bind most patient isolates. Adhesion to endothelium devoid of CD36 may be possible using platelets expressing CD36 as a bridging interaction [16]. The carbohydrate CSA is uniquely implicated in placental adhesion during pregnancy. Parasites able to adhere to CSA express an unusually conserved PfEMP-1 which is expressed during malaria in pregnancy [17].

It is known that cytoadherence of iRBC induces signalling events in endothelial cells [18,19]. Some of these signalling responses may help protect the host endothelium against damage, as suggested by the down-regulation of apoptotic genes in endothelial cells [20]. However, other effects may be damaging. For example, the induction of ICAM-1 expression on endothelial cells occurs in response to iRBCs [20], which could lead to further sequestration of iRBC, as well as local leukocyte recruitment, amplifying pathology caused by adhesion or local inflammatory responses. There is also evidence of induction of apoptosis and leakage in endothelial cells following adhesion of iRBC [21].

Antimalarial treatments are effective at killing parasites where resistance has not developed; however, there is still a high mortality rate associated with severe malaria with most deaths occurring in the first 24 h after hospital admission [22]. It is clear that even after initiation of antimalarial treatment, patients continue to manifest worrying clinical signs [22]. It has been suggested that iRBC after antimalarial treatment may still continue to cytoadhere—as observed in cerebral ultrastructural studies [23]. Furthermore a potential mechanism for the difference in effectiveness of artesunate versus quinine was suggested to be potentially due to activity in preventing sequestration [24]. However, it has not been shown using in vitro assays to known receptors how antimalarial treated iRBC can cytoadhere. We test these hypotheses, that non-viable parasites killed by standard antimalarial treatment contribute to disease pathology by their continued ability to cytoadhere. To investigate this phenomenon, we have performed in vitro assays for cytoadherence with drug-treated *P. falciparum* iRBCs to ICAM-1 and human endothelium under both static and flow conditions.

Using a range of antimalarials we show that non-viable *P. falciparum* iRBCs retain the ability to cytoadhere at least 24 h after drug administration as a result of the slow rate of degradation of the surface protein PfEMP-1.

## 2 Materials and methods

### 2.1 Parasite strains and culture

Parasite lines ItG [25] and A4 [26] were used in this study. Both of these strains are of the IT lineage and are well characterized for their ability to bind to ICAM-1 and CD36 [14]. ItG was selected on ICAM-1 protein and expression of the expected *var* gene confirmed by cloning and sequencing a DBLα tag from cDNA. A4 was selected for binding to the monoclonal antibody BC6 [27] followed by immunofluorescence analysis (IFA) of expressed PfEMP-1 and sequencing of the expressed *var* tag. Populations used were >80% homogeneous for the expected *var* tag. Parasites were cultured under standard conditions in RPMI 1640 medium (supplemented with 37.5 mM HEPES, 7 mM d-glucose, 6 mM NaOH, 25 μg/ml gentamicin sulphate, 2 mM l-glutamine and 10% human serum) at a pH of 7.2, in a gas mixture of 96% nitrogen, 3% carbon dioxide and 1% oxygen [28] and synchrony maintained using sorbitol lysis at ring stages [29]. Two cultures were maintained in parallel 24 h apart to allow stage matched controls for 24-h time point experiments.

### 2.2 Qualitative and quantitative measurement of PfEMP-1 levels of the surface of *P. falciparum* A4 iRBCs

PfEMP-1 on the surface of live A4 iRBC was visualised by immunofluorescence using BC6 monoclonal primary antibody (1:50 dilution) which specifically recognises an exposed epitope of the A4var41 PfEMP-1 [27]. This was followed by a secondary rabbit anti-mouse antibody (serotec) (1:100) followed by Alexa488 conjugated goat anti-rabbit antibody (molecular probes) (1:5000). All washes and antibody dilutions were in Dulbecco's PBS with 1% BSA and each incubation was at 37 °C for 30 min. Infected cells were stained with ethidium bromide (1 μg/ml), mounted on a microscope slide and analysed by confocal microscopy using Zeiss LSMPascal microscope and Zeiss Pascal and Photoshop software (Adobe). For flow cytometry analysis staining was carried out as above then infected cells were diluted in PBS and 500,000 events (red blood cells) acquired on Beckman Coulter FACS XL, gated using forward scatter and side scatter to acquire red blood cell populations excluding debris and then FL-1 and FL-2 fluorescence intensity measured. Analysis was performed using WinMDi software. Regions were drawn on density plots (i.e. Fig. 4B) of uninfected controls to gate uninfected cells such that <0.1% of the population fell into the region classed as ethidium bromide positive. This region was then used to create a histogram. Region R1 was created for A4var41 positive cells based on negative antibody controls, <0.1% of the ethidium bromide positive population fell into region R1 when no primary antibody was included. Similarly for the antigenically distinct strain ItG <0.1% of the ethidium bromide positive population fell into region R1 (not shown). The mean fluorescence intensity (MFI) of region R1 was used to quantitate the level of PfEMP-1 on A4var41 positive infected red blood cells. The percentage of A4 iRBC that were classed as A4var41 positive remained at ∼80%.

### 2.3 Endothelial cells

Human umbilical vein endothelial cells (HUVEC) and human dermal microvascular endothelial cells (HDMEC) were purchased from Promocell and cultured as per manufacturer's instructions. Cells at passages 4–6 were used for all experiments. Prior to experimentation cells were stimulated by the addition of 1 ng/ml TNF for 18 h. This procedure allowed for the induction of ICAM-1 expression on the surface of the endothelial cells as verified by immunofluorescence microscopy and flow cytometry after immunofluorescence (data not shown).

### 2.4 Adhesion assays

All adhesion assays were carried out essentially as previously described [14]. In brief:

### 2.5 Flow based protein assays

Aminopropyltriethoxysilane (APES) treated microslides were coated with ICAM-1-Fc [30] protein at 50 μg/ml in Dulbecco's PBS. Protein was allowed to adhere for 2 h, then slides were washed with 1% BSA/PBS and blocked in 1% BSA/PBS overnight at 4 °C. Parasites were prepared to 3% parasitaemia and 1% haematocrit (hct) in binding buffer (RPMI 1640 medium pH 7.2, supplemented with 6 mM glucose). After flowing binding medium through the microslide for 2 min, prepared parasites were flowed through at a pump speed of 0.186 ml/min, to give a wall shear stress of 0.05 Pa (as previously used [14]) for 5 min. This was followed by flowing binding buffer for 2 min. All assays were carried out at 37 °C. Stationary adhered parasites were counted in six fields along the slide. Each experiment was performed in duplicate or triplicate and on three independent occasions. Results shown are compared to 0 h control culture, however an untreated control at equivalent mid-trophozoite-stage was used with each time point and showed no significant difference in cytoadherence compared to 0 h control.

### 2.6 Static endothelial cell assays

Endothelial cells cultured as above were seeded onto 13 mm thermanox coverslips (Nunc) coated with 1% gelatin in 24-well plates. Confluent cells were induced with 1 ng/ml TNF for 18 h and washed in culture medium before an assay. Parasites were prepared to 1% hct and 3% parasitaemia in binding buffer. 0.5 ml of cell suspension was applied to each well and incubated at 37 °C for 1 h with gentle resuspension by rotation every 10 min. Unbound cells were removed by two washes in binding buffer followed by 2× 30 min gravity washes, in which coverslips were balanced cell side down on 0.5 ml binding buffer in a 24-well plate at a 45° angle to allow for removal of cells not firmly adherent. Cells were fixed with 1% glutaraldehyde for ≥1 h. After staining with 5% Geimsa for 30 min, coverslips were washed in water, air dried and mounted using DPX hard set mounting medium. 6–10 areas of each cover slip were counted for the number of bound parasites. Each experiment was performed in duplicate or triplicate and on three independent occasions. Results shown are compared to 0 h control culture, however an untreated control at equivalent mid-trophozoite-stage was used with each time point and showed no significant difference in cytoadherence compared to 0 h control.

### 2.7 Flow based endothelial cell assays

APES treated microslides were coated with 1% gelatin, 1% collagen for 45 min at 37 °C. Endothelial cells at passages 3–5 were treated with trypsin to detach adherent cells (Promocell detach kit used as per manufacturers’ instructions). Cell suspensions were applied to the microslides, and allowed to settle for 2 h at 37 °C to form a confluent monolayer. After overnight growth with media exchange every hour, TNF (1 ng/ml) was added to the confluent cells for 18 h before the assay. Parasites were prepared at 3% parasitaemia and 1% hct in binding buffer. After flowing binding medium through the microslide for 2 min, prepared parasites were flowed through at a wall shear stress of 0.05 Pa for 5 min, followed by a wash with binding buffer (2 min). Stationary adherent parasites were counted in at least six fields along the slide. Each experiment was performed in duplicate or triplicate and on three independent occasions. Results shown are compared to untreated control culture at equivalent mid-trophozoite-stage for each time point.

### 2.8 Drug treatment

The effect of parasite viability on cytoadherence was tested using a range of drugs including artesunate, quinine, lumefantrine and piperaquine. Final drug concentrations were chosen based on published peak plasma levels. These were: 500 nM for artesunate [31,32], 25 μM for quinine [33,34], 15 μM for lumefantrine [35,36], and 100 nM for piperaquine [33,37].

The concentrations of drugs used were well above the IC_50_ determined for each (Table 1, supplementary information) and were confirmed to inhibit parasite development as assessed by Geimsa staining as described in Section 3. Parasites were confirmed non-viable after treatment by continued culture for at least 72 h post-treatment with no growth. The minimum exposure time to 500 nM artesunate to stop parasite growth was determined to be 30 min. Various exposure times greater than this followed by washing out of the drug did not affect binding phenotype 24 h following treatment (Supplementary Fig. S1). Drugs were washed out of PRBC cultures immediately prior to adhesion assays (after appropriate exposure time as indicated for each experiment) by 2× 5 ml washes in binding buffer before resuspension of culture in binding buffer. Parasitaemia and haematocrit were counted after drug exposure before adhesion assays and parasite suspension adjusted to 3% parasitaemia and 1% haematocrit for each assay.

Growth inhibition (IC_50_) assays were performed on ItG and A4 parasites using a standard fluorimetric assay [38,39].

### 2.9 Statistical analysis

Each binding experiment was performed in duplicate or triplicate and the mean calculated. Results shown are the mean of three independent experiments ±SD. Statistical significance was calculated using a two-tailed *t*-test.

## 3 Results

### 3.1 Cytoadherence of artesunate treated *P. falciparum* ItG iRBCs to ICAM-1 under flow

**Fig. 1:**
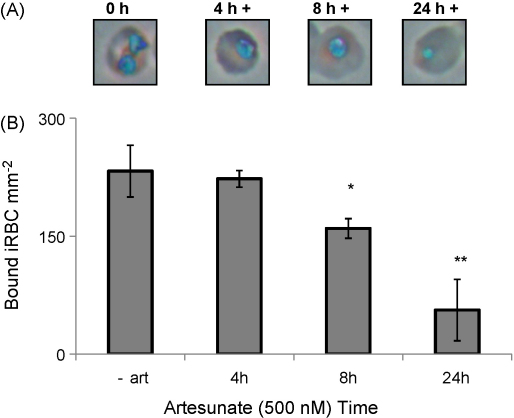
Phenotype of trophozoite-stage ItG iRBC after artesunate treatment. (A) Geimsa stained smears of ItG infected red blood cells after 0, 4, 8, and 24 h treatment with 500 nM artesunate under culture conditions. (B) Adhesion to ICAM-1 protein (50 μg/ml) under flow conditions (0.05 Pa shear stress) after 0, 4, 8 or 24 h treatment with 500 nM artesunate. Mean of three independent experiments ± standard deviation (SD). *P < 0.05 and **P < 0.01 compared to untreated control (c) trophozoites.

Adhesion of *P. falciparum* ItG iRBC to ICAM-1 protein under flow was assayed following 4, 8 and 24 h artesunate (500 nM) treatment of trophozoites, 24 h post-invasion (hpi) stage parasites. Parasite development as assessed by Geimsa staining appeared to halt after 4 h treatment (Fig. 1A) and parasites were noticeably pyknotic following 8 h treatment. As described in Section 2, parasites were not viable for culture following drug treatment.

Cytoadherence of iRBC occurs during the second part of the 48 h intraerythrocytic cycle of the parasite, and no significant changes in the level of cytoadherence of untreated, developing iRBC occur after adhesion reaches a maximum at 24–26 h post-invasion (not shown). After drug addition to 24 hpi iRBC no significant difference in binding was observed for parasites exposed to artesunate for 4 h compared to untreated parasites (Fig. 1B). Following 8 h exposure, a slight reduction in binding was seen and following 24 h artesunate treatment adhesion to ICAM-1 was reduced to ∼30% of control levels (*P* < 0.01, Fig. 1). The concentration of artesunate used (500 nM) was chosen as it falls in the normal range reported for observed plasma concentrations (see Section 2 and Supplementary Table 1), however, similar binding phenotypes were observed for artesunate concentrations ranging from 20 nM to 12.5 μM (Supplementary Fig. S2).

### 3.2 Cytoadherence of artesunate treated *P. falciparum* ItG iRBCs to human endothelial cells

Adhesion of *P. falciparum* ItG iRBCs to human endothelial cells (HUVEC and HDMEC) was assessed under both static and flow conditions following 4, 8 and 24 h artesunate (500 nM) treatment of trophozoite (24 h post-invasion) stage parasites.

As described in Section 2, binding assays were performed with confluent monolayers of TNF (1 ng/ml, 18 h) activated endothelial cells. Under these conditions HUVEC express the receptor ICAM-1, whereas HDMEC express both CD36 and ICAM-1.

**Fig. 2:**
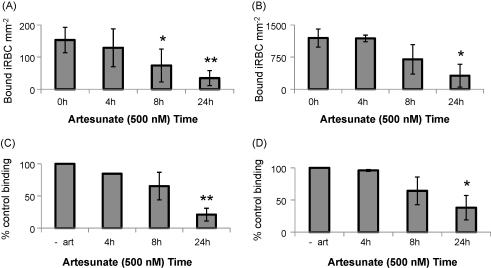
Adhesion of trophozoite-stage ItG iRBC to endothelial cells after 0, 4, 8 or 24 h artesunate treatment. Binding under static conditions to (A) HUVEC and (B) HDMEC after TNF stimulation (1 ng/ml, 18 h) showing bound iRBC per mm^2^ confluent endothelial cells. Binding under flow conditions to (C) HUVEC and (D) HDMEC after TNF stimulation of endothelial cells showing percent of control (untreated trophozoites) binding. Each represents mean of three independent experiments ± SD. *P < 0.05 and **P < 0.01 compared to untreated controls (c).

Under static conditions, there was no significant difference in the level of binding to either HUVEC or HDMEC of *P. falciparum* ItG iRBCs incubated for 4 h with artesunate (500 nM) compared to the untreated controls to (Fig. 2A and B). Following 8 h exposure, a slight reduction in binding was seen and following 24 h artesunate treatment adhesion to both HUVEC (*P* < 0.01) and HDMEC (*P* < 0.05) was reduced to ∼30% of control levels (Fig. 2A and B).

Essentially the same binding phenotypes were observed for adhesion measured under flow, with up to 8 h exposure to artesunate (500 nM) not significantly affecting adhesion to both HUVEC or HDMEC, whilst 24 h exposure to artesunate (500 nM) did reduce the levels of binding to both cell types to 25–30% of control levels (Fig. 2C and D).

### 3.3 Cytoadherence of other *P. falciparum* isolates to other receptors after artesunate treatment

Similar results to those seen for ItG adhesion after artesunate treatment were also seen with two other parasite isolates. A4, like ItG, binds to both ICAM-1 and CD36 but generally at lower levels than ItG [14]. The adhesion of A4 to ICAM-1 protein under flow and to HDMEC under static conditions was assessed. No significant reduction in adhesion was seen after 8 h treatment of trophozoite-stage parasites with artesunate (500 nM), however, after 24 h treatment adhesion was reduced to 25–30% of controls. Additionally the CSA binding strain CS2 [40] was assayed for cytoadherence to CSA protein under flow conditions. No reduction in adhesion was seen after 8 h treatment and significant adhesion at around 25% control levels was observed after 24 h treatment. Experiments after 4 and 24 h drug treatment showed that ItG binding to CD36 was similarly affected (Supplementary Fig. S3).

### 3.4 Measurement of PfEMP-1 levels on the surface of drug-treated *P. falciparum* iRBCs

To determine whether the continued ability of non-viable parasites to adhere is due to the persistence of the PfEMP-1 complex on the infected erythrocyte membrane, single cell and population immunofluorescence measurements were performed using the monoclonal antibody mAb BC6, specific for the a surface epitope of the PfEMP-1 A4var41 variant [26].

**Fig. 3:**
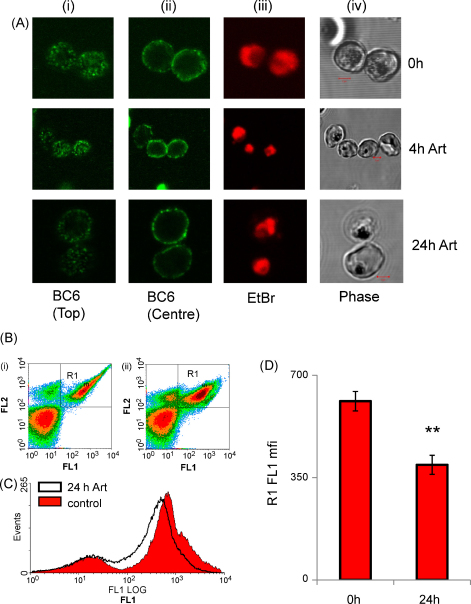
Analysis of PfEMP-1 on the surface of live trophozoite-stage A4 iRBC after immunofluorescence using the primary antibody BC6. (A) Confocal fluorescence images of (i) top plane, and (ii) centre plane of BC6 fluorescence, (iii) ethidium bromide labelling of parasite, and phase contrast images (iv), after 0 (upper) 4 (middle) or 24 h (lower) artesunate treatment. Scale bar (2 μM) on phase contrast image. (B) Quantitation of PfEMP-1 levels by flow cytometry analysis after BC6 primary antibody and Alexa488 coupled secondary antibody (FL-1, *X*-axis) and counterstaining infected red blood cells with ethidium bromide (FL-2, *Y*-axis). In the density plots uninfected red blood cells are the population in the lower left. Box R1 represents BC6 positive infected red blood cells. Plot (i) shows 0 h culture, plot (ii) shows the culture after 24 h artesunate treatment. (C) Histogram showing quantitation of BC6 positive fluorescence intensity (*X*-axis) of the infected population (gated as ethidium bromide positive) untreated trophozoites (open black) or after artesunate (500 nM) 24 h (solid red). (D) Mean fluorescence intensity (MFI) for PfEMP-1 positive population (gate R1) mean ± SD for three independent experiments, ** indicates a statistically significant difference compared to controls (*P* < 0.01) as determined by *T*-test. (For interpretation of the references to color in this figure legend, the reader is referred to the web version of the article.)

Confocal laser scanning microscopy of *P. falciparum* A4 iRBCs after immunofluorescent labelling with the primary monoclonal antibody BC6 revealed the characteristic punctate distribution of PfEMP-1 on the outer erythrocytic membrane (Fig. 3A). Qualitatively, the distribution of PfEMP-1 was not observably different in *P. falciparum* iRBCs treated for up to 24 h with 500 nM artesunate (Fig. 3A). Analysis by flow cytometry confirmed that ∼80% of iRBCs expressed detectable levels of A4var41 PfEMP-1 (Fig. 3B). This population percentage did not change through the course of these experiments. Following treatment with artesunate (500 nM, 24 h), the mean fluorescence of PfEMP-1 surface positive iRBCs was reduced to approximately 65% the level of untreated cells (*P* < 0.01, *n* = 3, Fig. 3C and D). Notably, PfEMP-1 could still be detected on iRBC surface after 4 days treatment (data not shown).

### 3.5 Cytoadherence of *P. falciparum* ItG iRBCs to ICAM-1: comparison of antimalarials

Adhesion of *P. falciparum* ItG iRBC to ICAM-1 protein under flow was assayed following 4, 8 and 24 h drug treatment of trophozoite (24 h post-invasion) stage parasites. The antimalarial drugs tested were artesunate (500 nM), quinine (25 μM), lumefantrine (15 μM) and piperaquine (100 nM). As described in Section 2, all drugs were used at concentrations comparable to published peak plasma concentrations and were confirmed to result in the abrogation of parasite viability.

**Fig. 4:**
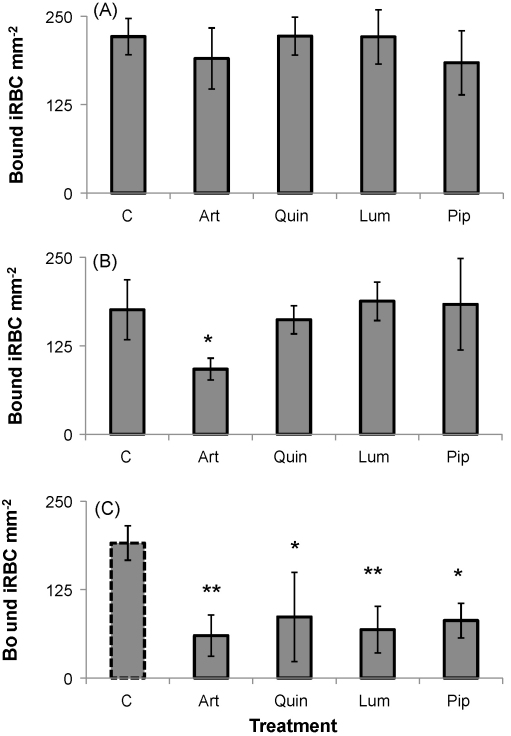
Adhesion of trophozoite-stage ItG iRBC to ICAM-1 protein under flow conditions after 4 h (A), 8 h (B) and 24 h (C) treatment with artesunate (art) (500 nM), quinine (quin) (25 μM), lumefantrine (lum) (15 μM) or piperaquine (pip) (100 nM). Results are mean of three independent experiments ± SD. *P < 0.05 and **P < 0.01 as determined by *T*-test compared to untreated control (c) at each time point. The untreated control for the 24 h time point was a stage matched untreated mid-trophozoite culture indicated by a dotted outline as the untreated original culture had reinvaded to non-cytoadherent ring stages. There was no significant difference in the adhesion of any of the untreated controls at the different time points. Treated cultures were dead pyknotic “trophozoite-stage” parasites.

The binding phenotypes for all treatments were observed to be very similar, with no significant difference in binding observable after 4 h drug exposure (Fig. 4A). As observed previously, a small reduction in binding was observed for parasites exposed to 8 h artesunate (Fig. 4B) but no significant difference relative to control cells was observed for all other drugs (Fig. 4B). A significant reduction in iRBC binding was observed following 24 h exposure to all of the drugs, with binding ∼30% of control levels (Fig. 4C).

### 3.6 Cytoadherence of *P. falciparum* ItG iRBCs to ICAM-1: comparison of antimalarials administered to ring stage parasites

As the antimalarial drugs used in this study have different pharmacodynamic properties, especially in regards to cell cycle specificity, binding experiments to ICAM-1 under flow conditions were repeated as described above but drug exposure was performed on ring stage (12 h post-invasion) parasite cultures. These younger ring stage parasites represent the pre-cytoadherent circulating population.

**Fig. 5:**
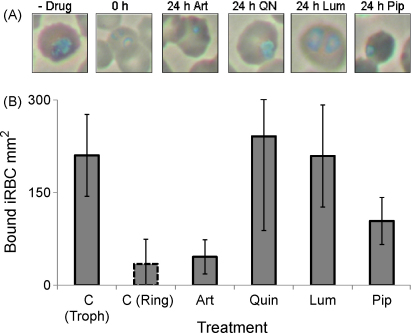
(A) Geimsa stained smears of ItG iRBC 24 h after the addition of indicated drug treatment to ring stage (young) parasites. (B) Adhesion to ICAM-1 protein under flow conditions 24 h after addition of treatment to ring stage (young) parasites. The untreated ring stage control (representing the starting culture) shown at the 24 h time point was a stage matched untreated culture indicated by a dotted outline as the untreated original culture had matured to the cytoadherent trophozoite-stage. A *P*-value <0.05 was obtained for the comparison between artemisinin treated parasites and control trophozoites.

Adhesion to ICAM-1 protein under flow, was determined after 24 h exposure of ring stage *P. falciparum* ItG iRBCs to artesunate (500 nM), quinine (25 μM), lumefantrine (15 μM) and piperaquine (100 nM) relative to untreated control cells. Significant differences were observed between the various treatments. Artesunate treated parasites were unable to mature to trophozoites and non-viable ring parasites were observed after 24 h exposure (Fig. 5A), whilst quinine and lumefantrine treated parasites matured to late trophozoites before dying and appearing pyknotic (Fig. 5A). Piperaquine treated parasites appeared to show an intermediate amount of development to late ring/early trophozoite before becoming non-viable. Binding experiments revealed that control parasites allowed to develop for 24 h from ring stage parasites to trophozoites displayed characteristic levels of adhesion whilst control ring stage parasites, as expected, displayed a very low level of cytoadherence (Fig. 5B). Artesunate treated parasites, which as described had failed to mature showed little cytoadherence, whilst quinine and lumefantrine treated parasites displayed high levels of adhesion comparable to control trophozoite-stage iRBC (Fig. 5B). Treatment of parasites with piperaquine resulted in intermediate levels of binding, ∼50% of control levels (Fig. 5B).

## 4 Discussion

In this study we have assessed the ability of non-viable parasites killed by antimalarial treatments to cytoadhere, a process important in the pathophysiology of disease.

Our data demonstrate that *P. falciparum* iRBC are capable of cytoadherence for at least 24 h after lethal dosages of a range of antimalarial drugs. The antimalarial drugs tested in this study were artesunate, quinine, lumefantrine and piperaquine at clinically relevant concentrations, chosen to mimic peak plasma drug levels. The cytoadherence phenotype of iRBCs was measured using both static and flow assays and using a range of parasite strains (ItG, A4 and CS2) against purified receptor proteins (ICAM-1, CD36 and CSA) and endothelial cells (HUVEC and HDMEC).

These observations are consistent with observations from a previous ultrastructural analysis of brains from patients with cerebral malaria [23] who suggest that they observe morphologically damaged parasites adhered to cerebral endothelium, consistent with parasite death in situ and maintained adhesion.

Consistent with the continued ability to cytoadhere, we observed the characteristic punctate distribution of PfEMP-1 on the surface of *P. falciparum* iRBC at significant levels (∼65% of control) following 24 h antimalarial treatment (Fig. 3).

These data illustrate the stability of the changes made to the red blood cell by the parasite such that the remodelling of the infected red blood cells is maintained after the parasite is rendered non-viable. Nevertheless, quantification of the PfEMP-1 levels on iRBCs surface showed some reduction after drug treatment, consistent with a degree of degradation/loss of the protein. This reduction in PfEMP-1, combined with potential reduction in other cytoadherence-related parasite proteins, i.e. KAHRP, could explain the reduced levels of cytoadherence seen after 24 h treatment. This could be comparable to that seen when mutations are present in haemoglobin, i.e. haemoglobin C (HbC), and sickle haemoglobin (HbS). In these cases infected erythrocytes show a slightly reduced and abnormal display of PfEMP-1 and concurrently reduced cytoadherence [41].

Testing a selection of antimalarial drugs showed that the effect on mature (24 h post-infection) trophozoite cytoadherence was not drug specific; the same level of adhesion was seen after 24 h treatment with all of the antimalarials tested (Fig. 3).

Previously published data has implied that antimalarial drugs including quinine, halofantrine, artesunate and artemether reduce cytoadherence and/or rosetting in *P. falciparum* [42]. In the Udomsangpetch et al., study, parasites (presumably ring stage circulating parasites) were exposed to antimalarial drugs for 2–24 h, washed and grown in culture until control parasites (no drug exposure) were at late trophozoite/early schizont stage. Depending on the drug treatment these authors reported a range of binding phenotypes, most notably parasites exposed to artesunate and artemether were reported to result in ≥50% inhibition of both cytoadherence (static assay) and rosetting. It was concluded by these authors that the observed differences between the various drugs on parasite adhesion probably reflected differences in the susceptibility of asexual stage parasites to antimalarials.

We show a similar effect when drug treatment is added to ring stage (young) parasites. As shown in Fig. 5A, the various antimalarials used inhibited parasite growth at different stages of the asexual cycle. This is consistent with previous pharmacodynamic data on the mode of action of these drugs. The minimum exposure time to artesunate (500 nM) to render parasites non-viable was just 30 min, reflecting the fast mode of action of this drug upon activation of the endoperoxide bridge [43]. Furthermore the drug is known to be active against young ring stage parasites. The activity of lumefantrine, quinine and piperaquine, however, is based on the disruption of haeme detoxification [44,45] occurring later in development during haemoglobin digestion by trophozoite-stage parasites [43,46,47]. The pharmacodynamic differences of these drugs when administered to ring stage parasites results in mismatched comparisons of binding phenotypes. Essentially the assays measured the adhesion of dead ring stage parasites for the artesunate treated group, against the adhesion of dead trophozoite-stage parasites for the quinine, lumefantrine and piperaquine treated groups (Fig. 5A and B). The significant reduction in adhesion seen by artesunate treated parasites in Fig. 5 and in the Udomsangpetch et al., study [42] simply reflect that these groups consist of ring stage parasites that have insufficient levels of binding proteins, i.e. PfEMP-1, exported to the erythrocyte membrane surface at that stage.

The significance of this difference between ring stage acting artemisin based treatments and treatments that only act on mature parasites (i.e. quinine), has also been discussed in the context of the seaquamat trial. This trial compared artemisinin and quinine for the treatment of severe malaria in adults and showed an advantage for artesunate over quinine, particularly in groups with high parasitaemia [24]. The authors suggest that this advantage may stem from the mechanism of action of artesunate on immature parasites thereby preventing the next round of sequestration. Our in vitro results support that this hypothesis could be valid.

The significant observation of our study is that dead parasites retain the ability to cytoadhere irrespective of the antimalarial treatment administered. However, we can hypothesise that administration of fast, ring stage acting antimalarials such as artesunate will result in a reduction of *de novo* cytoadherence when administered in vivo due to the their ability to prevent maturation of parasites to mature cytoadherent stages. The persistence of cytoadherence for many hours after treatment, despite rapid effects on parasite viability fits with, and may in part explain, the clinical observation of a high mortality rate in first 24 h after hospital admission after initiation of antimalarial treatment [22]. Even though treatment is killing the parasites rapidly, we show that they would still be able to cytoadhere 24 h following treatment, potentially still contributing to disease pathology.

It is conceivable that the observed phenomenon of continued adherence by non-viable *P. falciparum* iRBCs may be targeted by adjuvant therapies designed to prevent or preferably reverse cytoadherence. Indeed, studies have recently been performed with Levamisole, which was shown to reduce sequestration of *P. falciparum* in patients by inhibition of CD36 dephosphorylation [48].

In summary, this study has assessed the cytoadherence capability of drug-killed *P. falciparum* iRBCs. Our data demonstrate that non-viable parasites, irrespective of treatment retain the ability to cytoadhere for up to 24 h. This phenomenon is due to the stability of the changes made to the host red blood cell by the parasite, as illustrated by the continued presence of PfEMP-1 on the surface of iRBC after loss of parasite viability. This continued capacity for cytoadherence could contribute to disease pathology following treatment. It is hypothesised that fast and ring stage acting antimalarials such as the endoperoxides may be therapeutically more favourable by their ability to prevent *de novo* adhesion, but adjunctive therapy strategies that can also reverse cytoadherence may also be advantageous in addressing the high mortality seen in severe malaria early after hospital admission.
